# Current Challenges and Future Directions of Research in Cell Phone Addiction

**DOI:** 10.1192/j.eurpsy.2024.616

**Published:** 2024-08-27

**Authors:** O. Vasiliu

**Affiliations:** Psychiatry, Dr. Carol Davila University Emergency Central Military Hospital, Bucharest, Romania

## Abstract

**Introduction:**

Behavioral addictions (BAs) are intensely explored during the last decades due to their impact on the quality of life, functionality, socio-economical negative consequences, and high risk of mental health negative consequences. BAs are new challenges for clinicians and researchers due to a lack of well-defined diagnostic criteria, very few available epidemiological data, and scarce information about efficient therapeutic interventions. Cell phone addiction (CPA) has been raising a significant interest for mental health specialists because of its increasing prevalence and potential long-term physical and mental complications. Therefore, an analysis of the available data about the main characteristics of this pathology seems granted.

**Objectives:**

The main objective of this review was represented by the need to find relevant reports about the epidemiological, clinical, and therapeutic interventions in CPA.

**Methods:**

A narrative review focused on the available treatments for food addiction was performed through a search in four electronic databases (PubMed, Cochrane, EMBASE, and Web of Science/Clarivate) using the paradigm “cell phone addiction” or “smartphone dependence” and “treatment” or “epidemiology” or “diagnostic criteria” or “risk factors.” No inferior time limit for published papers was established, and the superior limit was July 2023.

**Results:**

A relatively large number of papers regarding this topic were found (n=772), but after applying the inclusion and exclusion criteria, only 29 articles remained. Female gender and adolescents, but also high anxiety levels, insomnia, excessive Internet use, less physical activity, and a higher level of dependence have been correlated with CPA. Six validated scales have been identified as possible instruments for monitoring the CPA evolution. Different diagnostic criteria have been suggested, but they still lack clinical validation. Cognitive-behavioral therapy could be helpful, and smartphone applications that limit online time could also be efficient. Treatment of previously mentioned vulnerability factors is also recommended to obtain long-term favorable effects.

**Conclusions:**

CPA is an increasingly explored BA, but validated diagnostic criteria are still missing. The treatment is also based on extrapolations from other addictions. Therefore large sample-based therapy trials are needed.

**Disclosure of Interest:**

None Declared

